# Seasonal Colony Loss Rates and Honey Bee Management in the Kingdom of Saudi Arabia: Results of a Survey of Beekeepers

**DOI:** 10.3390/insects14060513

**Published:** 2023-06-01

**Authors:** Abdulmajeed Barrak Albarrak, Alison Gray

**Affiliations:** 1 Department of Mathematics and Statistics, University of Strathclyde, Glasgow G1 1XH, UK; 2 Mathematics Department, College of Science, Jouf University, Sakaka P.O. Box 2014, Saudi Arabia; aalbarrak@ju.edu.sa

**Keywords:** honey bee, face-to-face survey, online survey, colony loss, seasonal loss rates, beekeeping practice, Saudi Arabia, *Varroa destructor*

## Abstract

**Simple Summary:**

Relatively little is known about experience of beekeepers, honey bee management practices and colony loss rates in Saudi Arabia and the Middle East compared to other countries, yet beekeeping and honey production are important in the Saudi economy. Results of a survey of 109 beekeepers in Saudi Arabia conducted in 2018 are presented, from a purpose-designed questionnaire, including colony losses over 5 seasons, to help fill this knowledge gap. The participants were managing 135 to 1700 colonies and had 2 to 45 years of beekeeping experience. Most (73.1%) respondents were mainly keeping local hybrid bees, while 25.9% mainly kept the Yemeni honey bee. Most (83.5%) beekeepers reported losing colonies over the time period studied. The reported colony loss rate was significantly higher in summer than in other seasons: the overall proportion of colonies lost was 11.4% in summer 2017 and was lowest in spring 2018 (6.6%). The main reported causes of colony losses were the Varroa mite, then disease. However, the bee wolf was the major named pest. Honey produced per colony varied greatly between beekeepers rather than bee races. The results establish a benchmark for future beekeeper surveys in Saudi Arabia and other environmentally similar countries where colony losses are of interest in all seasons of the year.

**Abstract:**

There is high demand for honey in Saudi Arabia, honey bees make a valuable contribution to agriculture and the economy, and therefore it is important to know levels of colony loss and potential reasons for losses. While there is much research into honey bee colony losses worldwide, little is known about colony losses in Saudi Arabia, management practices or beekeeping experience there. The aims of this work were to address this knowledge gap. Results of a survey of beekeepers in southwest Saudi Arabia conducted in summer 2018 are presented, including colony losses in five different seasons. Data collection involved face-to-face interviews, supplemented by an online survey, using a purpose-designed questionnaire. Responses were obtained from 109 beekeepers, all male, managing 135 to 1700 colonies, with 2 to 45 years of beekeeping experience. Most (73.1%) respondents mainly kept local hybrid bees, while 25.9% mainly kept *Apis mellifera jemenitica*. Honey yields per colony varied much more between beekeepers than between bee races. A high proportion (83.5%) of beekeepers reported losing colonies over the period studied. The reported colony loss rate was significantly higher in summer than in other seasons, but still low. The overall proportion of colonies lost was 11.4% in summer 2017 and was lowest in spring 2018 (6.6%). The main reported causes of loss were *Varroa destructor* and disease. Most beekeepers (88.0%) treated against the Varroa mite, although only one method was reported, tau-fluvalinate as Apistan strips, and only 41.7% used a screened bottom board. The results establish a benchmark for future beekeeper surveys in Saudi Arabia and other environmentally similar countries where colony losses are of interest in all seasons of the year. Informing and supporting Saudi beekeepers concerning Varroa monitoring and treatment and optimal hive management could result in fewer losses, higher honey yields, potential to market organic honey and a greater share of the domestic honey market.

## 1. Introduction

While many countries in recent years have been conducting surveys of beekeepers in order to study beekeeping practices, colony losses, and in particular to identify risk factors for colony loss [1,2,3,4,5,6,7], relatively little is known about beekeeping practices and colony losses in some parts of the world, including the Middle East [8]. In particular, little is known about rates of colony loss or causes of colony loss in Saudi Arabia. A few researchers have been very active in studying honey bees in Saudi Arabia, and an overview of the nature of beekeeping in Saudi Arabia was given in [9], but there are few studies of colony loss [10] and no systematic surveys of beekeepers. 

Since the large-scale colony losses experienced in the USA in winter 2006/2007 [11], and others observed later in Europe [12,13], leading to the establishment of the international research network COLOSS [12,14], intensive research efforts have been made to monitor levels of colony losses in many countries. Such annual monitoring has been a core activity of COLOSS since its inception, carried out through the COLOSS monitoring Core Group [1,15,16,17,18,19,20,21]. This monitoring involves a standardised survey questionnaire [22] and a network of national coordinators using this questionnaire, translated as necessary, in a survey of beekeepers in their own country. The use of a standardised survey, data checks and a common data analysis gives comparability of the calculated colony loss rates for the participating countries. Most countries in the COLOSS monitoring group are in Europe, where colonies are most susceptible to loss over the winter, and the active season is the spring–summer period, when losses are relatively rarer and opportunities are available for bee management and replacement of lost colonies. In countries or continents without a cold winter, colony losses in other seasons are also a concern, for example in South America, Africa, Israel and parts of the USA [4,5,23]. In such countries, summer and annual losses are important. Algeria, Israel, Mexico and to a lesser extent Egypt, for example, have for some time participated in the COLOSS monitoring, e.g., [18,21], however, Saudi Arabia has not so far been involved. 

The aims of this work were to carry out a purpose-designed survey in Saudi Arabia to obtain a profile of beekeepers, their beekeeping management practices and experience and to evaluate colony loss rates in different seasons and causes of colony loss. The COLOSS colony loss monitoring survey questionnaire and related questionnaires were used as a basis for the questionnaire design. A related aim was to examine whether there were any differences in the seasonal loss rates. It was hypothesised that the loss rate could be affected by weather conditions, specifically the very dry and extremely hot conditions in summer [9]. In relation to this, questions were asked about the apiary environment, the hive type, and any migration of colonies. Honey yield and honey bee race were also recorded, as yield may differ between races. More general questions about colony management, including awareness of Varroa and control of the Varroa mite, were also used in the survey. To the best of our knowledge, this is the first such survey carried out in Saudi Arabia. 

## 2. Materials and Methods

### 2.1. Organisation of the Survey

**Figure 1:**
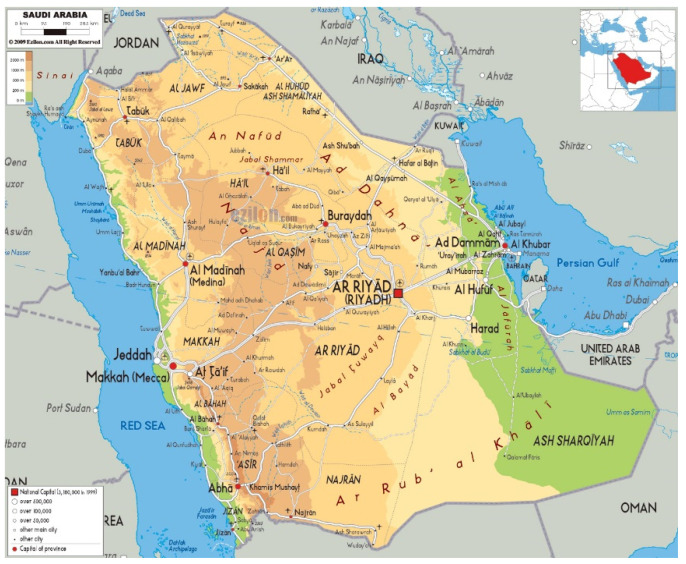
Map of Saudi Arabia, showing the southwestern mountain region. Source: http://www.maps-of-the-world.net/maps/maps-of-asia/maps-of-saudi-arabia/large-physical-map-of-saudi-arabia-with-roads-cities-and-airports.jpg (accessed on 18 May 2023); public domain image licensed under CC BY-SA 3.0.

Data collection was conducted through a face-to-face survey by the first author over a 3-month period, mid-May 2018 to mid-August 2018, to allow time for travel. Visits were made to beekeepers belonging to 7 regional beekeepers associations, identified via the Ministry of Environment, Water, and Agriculture, as the active associations. These are all in the south and west of the country, where most beekeeping is practised: Makkah (Mecca), Taif, AlBaha, Abha, Rijal Almaa, Jazan, and Najran [9,24]; see Figure 1. The head of each beekeeping association was contacted and visited, and they or their representative identified and provided details of beekeepers who could be contacted by phone.

This approach ensured participation of beekeepers in all the regions where the beekeeper associations were active in the southwest, therefore the result should be geographically representative of beekeeping in that part of the country. It also allowed the association representative to introduce the person conducting the survey to the beekeeper, if necessary.

Those beekeepers agreeing to participate were visited at an agreed time, region by region. A hard-copy questionnaire was completed at each visit, giving 98 responses. An additional 11 responses were obtained over several weeks through an online version of the questionnaire using Google Docs survey tools, publicised to beekeeper associations via WhatsApp.

Each participant signed or ticked an informed consent declaration before completing the survey, being informed concerning the nature and purpose of this scientific study and management and use of the data. They were assured of anonymity in the reporting of the results and no sensitive information was collected. The data were handled in line with the UK’s Data Protection Act and the General Data Protection Regulation.

### 2.2. The Questionnaire

A primary goal was to collect information on colony losses, as well as beekeeping practices generally. Therefore, a purpose-designed questionnaire was developed, adapted from the standardised questionnaire of the COLOSS colony loss monitoring Core Group, which is used in many countries [1,14,22]. Some questions were also included from a questionnaire used in colony loss monitoring in Algeria, as a participating country for many years in the COLOSS surveys and a hot country where it was expected that beekeepers would face similar challenges to those in Saudi Arabia. As this was, to our knowledge, a first survey of colony losses specifically in Saudi Arabia, the questions concerning colony losses and numbers of colonies managed were kept simple, to avoid difficulties in explaining or answering the questions. Other questions were added, and the final questionnaire was translated into Arabic. 

The first group of questions asked for background information on the beekeepers, their purpose, motivation and experience in beekeeping. The second group of questions concerned the number of apiaries managed and the environmental conditions in which colonies were kept. A further set of questions asked about numbers of colonies and lost colonies in each season and causes of any losses. The beekeeper was simply asked to state the number of colonies managed in each season and the number lost in that season, as in the COLOSS questionnaire (although the latter also considers different types of colony loss). The remaining questions related to bee management practices, monitoring and treatment of colonies against the Varroa mite. The questionnaire is included as Questionnaire S1 in Supplementary Materials.

#### Definition of the Seasons

The questions asked about numbers of colonies managed and lost in each of 5 seasons: spring 2017, summer 2017, autumn 2017, winter 2017–2018 and spring 2018. The seasons were defined by consideration of weather temperatures, as follows: Spring as March to April, Summer as May to August, Autumn as September to October, and Winter as November to February.

### 2.3. Data Processing and Statistical Analysis

Data recorded on hard-copy questionnaires from the visits to beekeepers were transferred later to an Excel spreadsheet using a consistent coding scheme, and electronic responses from the online survey were added to this. Due to the face-to-face nature of most of the data collection, there was little missing data and no inconsistent colony loss data (no beekeeper specified losing more colonies than they stated they had that season), so little data filtering was necessary. All data analysis was carried out using the R software [25]. Colony loss rates were estimated as the proportion of colonies lost by all the beekeepers in any one season, i.e., total number of colonies lost divided by the total number of colonies managed by the beekeepers in that season. The difference in seasonal loss rates was tested using a Chi-squared test of proportions. Confidence intervals for the loss rates were obtained from quasi-binomial generalised linear modelling using a logit link function, as has been found to be appropriate for such colony loss data [22] and as used in the COLOSS monitoring surveys, e.g., [21]. Tests of association in two-way frequency tables used Fisher’s exact test. A *p*-value of less than or equal to 0.05 was considered as statistically significant.

## 3. Results

### 3.1. Response Rates and Description of Beekeepers

#### 3.1.1. Response Rates

Table S1 shows responses by region. In total, 141 beekeepers were contacted to participate, and 98 (69.5% of contacts) took part, as well as 11 online participants. The number of contacts provided by the associations varied from 82.8% of members for Najran, the smallest beekeeping association, to 17.4% for AlBaha, the largest association. The response rate from those contacts varied by region, from 80.9% in Abha to a low 46.6% in Rijal Almaa, one of the smallest beekeeping associations. The numbers visited were similar (13 to 17) for each association except for Rijal Almaa (only 7). The online responses included 6 beekeepers from AlBaha, 2 from Makkah, 2 from Abha and 1 from Taif.

#### 3.1.2. Description of the Beekeepers and Their Purpose in Beekeeping

**Figure 2:**
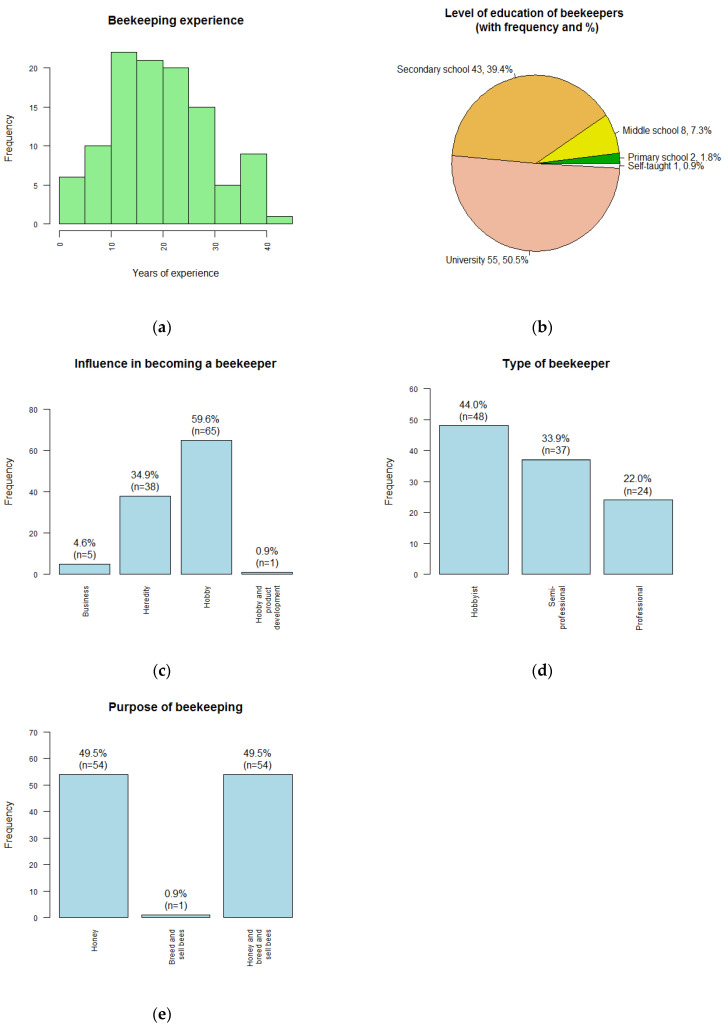
(**a**) Beekeepers’ years of experience; (**b**) Level of education; (**c**) Source of influence in becoming a beekeeper; (**d**) Type of beekeeper; (**e**) Purpose of beekeeping. Stated percentages may not add to 100% due to rounding error.

All contacted beekeepers were male. Their beekeeping experience ranged from 2 years to 45 years, with a median of 20 years. Most had 10 to 25 years of experience (Figure 2a), so were relatively experienced. Most of the 109 beekeepers were well educated; 50.5% had university-level education, most of the rest had secondary-level education (39.4%) (Figure 2b), and 10% had less education. They mainly described themselves as beekeepers (46.8%), with a further 5.5% saying they were businessmen. Most others were teachers (27.5%) or retired (11.9%).

Most (60.5%) of the 108 respondents answering became a beekeeper as a hobby or for a hobby as well as for hive products, while a substantial proportion (34.9%) had inherited their beekeeping operation, and only 4.6% said their motivation was business (Figure 2c). All stated the type and purpose of their beekeeping: 48 described themselves as hobby beekeepers (44.0%), 37 (33.9%) as semi-professionals whose beekeeping provides a substantial part of their income, and 24 (22.0%) as professionals whose beekeeping is their main income (Figure 2d). Therefore, more than half were semi-professional or professional beekeepers. Producing honey was a purpose for all except one beekeeper, while 50.5% also bred and sold bees (queens or nucleus colonies), which was therefore a major part of the commercial activity (Figure 2e).

### 3.2. Operation Size, the Apiary and Environment

#### 3.2.1. Operation Size

**Figure 3:**
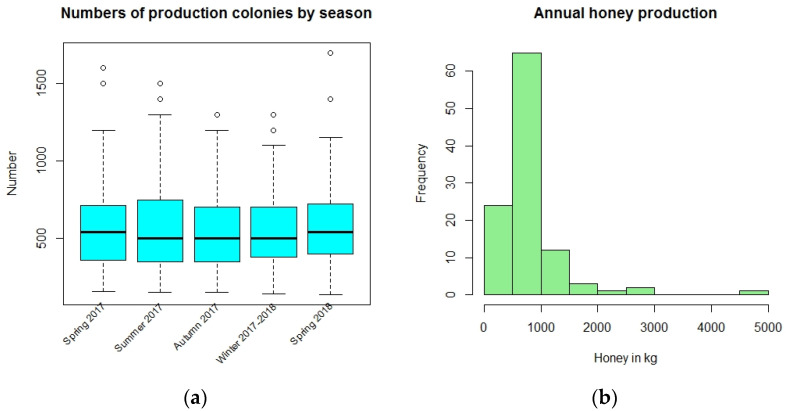
(**a**) Numbers of colonies across the seasons (the open circles mark unusual values); (**b**) Total annual honey production in kg per beekeeper in 2017.

All but one respondent had a single apiary, and the remaining respondent had 3 scattered apiaries, not all close (within 15 km) to each other. In every case the apiary or main apiary was kept in the region where the beekeeper lived. The number of production colonies ranged from 135 to 1700 and varied slightly across the seasons (Table 1 and Figure 3a). There was only one missing number of colonies, in winter. The number of colonies tends to be highest in spring. As these are large to very large-scale beekeepers, it is surprising that only one recorded having bees in more than one location.

#### 3.2.2. The Apiary Environment

Most respondents kept their apiaries in forest/woodland (67.6%), while almost all others (31.5%) kept them in cultivated fields (Table 2). Most locations used are shaded (63.9% of respondents) or sheltered (1 respondent), while the rest are sunny, corresponding well to the stated apiary environments. In extremely hot and dry conditions, a shaded environment is likely to be important for the well-being of bees. Beekeepers were asked separately about weather protection. Of the 108 respondents, 95 (88.0%) did protect their colonies against weather, 13 (12.0%) did not, and all of those taking measures to protect their colonies against weather used the “shaded” method. Further details were not provided, but it could partially relate to apiary location.

Migration of colonies is also reported here, as it is relevant not only for finding forage sources but also for obtaining suitable environmental conditions for better colony survival. Of the 109 beekeepers either answering whether or not they migrated their bees or giving a month of migration, 99 (90.8%) did practise migration. The location of migration was not specified, but 73 of 99 beekeepers (73.7%) migrated colonies in April and 26 (26.3%) in May, so migration is taking place in spring/very early summer before the weather becomes very hot. The reason for migration was not asked, but will almost certainly be for more adequate forage resources or more suitable weather conditions.

#### 3.2.3. Race/Subspecies of Bee

The great majority (79, 73.1%) of respondents reported that they mainly kept “local hybrids of no specific race”, while 28 (25.9%) mainly kept the Yemeni honey bee *Apis mellifera jemenitica*, and only one respondent said that he kept the Italian honey bee *A.m. ligustica.* Nobody was mainly keeping *A.m. carnica.*

#### 3.2.4. Honey Yield, Overall and by Subspecies

Annual total honey yield ranged from 200 kg to 5000 kg (Figure 3b), with a median yield of 800 kg (Table 3) over all colonies. Most production is between 500 kg and 1000 kg per operation, but several beekeepers reported exceptionally high output (Figure 3b). To compare yield from the different bee races, the total annual yield was divided by the average number of colonies over the seasons for each beekeeper, as a rough number of colonies managed, and yield per colony per beekeeper is given in Table 3.

Yield per colony is very variable, from 0.839 kg to 7.937 kg, and the largest yield is from local hybrids. Median (typical) yield is a little lower for the Yemeni bee (1.340 kg) than the local hybrid bees (1.404 kg) though the opposite is true for average yield. There is only one usable observation for the Italian bee, though its median yield per colony is slightly higher than from both local hybrid bees and *A.m. jemenitica*. The observed differences in yield per colony differ much more between beekeepers than between bee races and may reflect colony management.

### 3.3. Colony Losses

**Figure 4:**
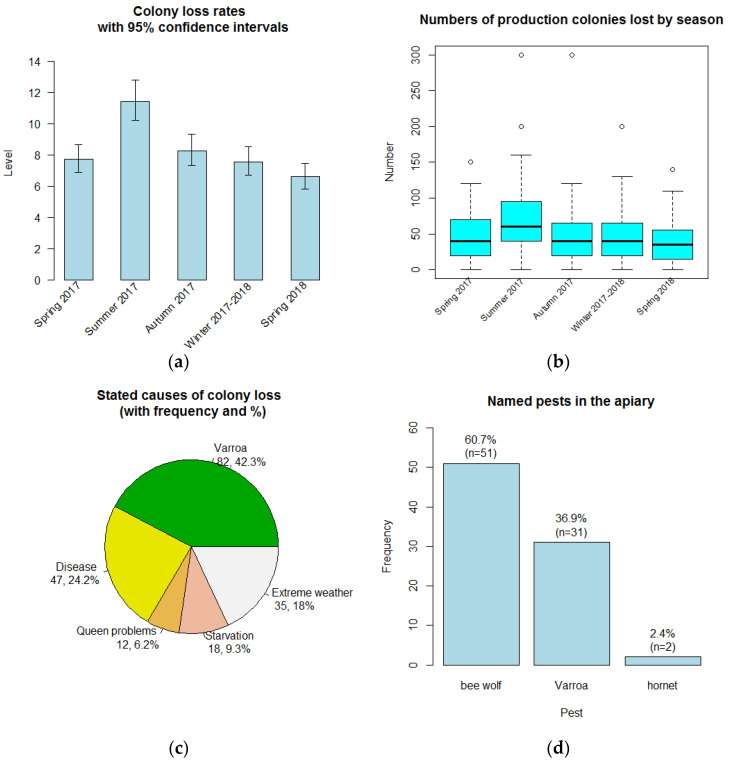
Colony losses: (**a**) Overall colony loss rates per season, with 95% confidence intervals; (**b**) Numbers of colonies lost across the seasons per beekeeper (the open circles mark unusual values); (**c**) Stated causes of colony losses; (**d**) Type of pests in the apiary.

Of the 109 beekeepers, 91 (83.5%) reported losing colonies, while 18 (16.5%) said they did not lose any. All except one of the beekeepers losing colonies lost colonies in every season, while one beekeeper did not lose any colonies in either spring season. There was one missing value for the number of colonies in winter 2017–2018, so that beekeeper was omitted from loss rate analysis for winter. Loss rates and their confidence intervals are shown in Table 4, separately for each season as colonies in each season will not necessarily all be different colonies. The loss rates differed significantly between seasons (Chi-squared test, *p* < 0.001). The loss rate in summer was significantly higher than in any other season (as judged by the non-overlapping confidence intervals for the summer loss rate and other loss rates; Table 4 and Figure 4a): 11.43% of colonies were lost in summer compared to 8.27% in autumn, 7.72% in spring 2017, 7.56% in winter 2017–2018 and 6.59% in spring 2018. There were some unusually high losses in summer 2017 and autumn 2017 (Figure 4b).

Beekeepers were able to state one or more than one reason, from a list, for their colony losses (Figure 4c). Of 194 reasons given by the beekeepers, Varroa was stated as the main reason for colony loss (42.3% of responses), then disease (24.2%). Extreme weather conditions accounted for only 18% of responses, while starvation (9.3%) and queen problems (6.2%) were relatively uncommon. Most of the 108 beekeepers answering (78.7%) had problems with pests in their apiaries, 16.7% said they did not, and a few (4.6%) did not know. Figure 4d shows that the bee wolf was the main pest, cited by 51 beekeepers, well above the Varroa mite (31 mentions), with 2 beekeepers mentioning hornets.

### 3.4. Colony Management

#### 3.4.1. Queen Replacement

Of 107 respondents, 91 (85.0%) did replace queens, while 16 (15.0%) did not. For those stating the reason for queen replacement, the main reason was in the case of loss of the queen (45 beekeepers; 49.5%), with both regular annual replacement and the case of a poor egg-laying queen being less common (23 beekeepers each; 25.3%). Nobody gave more than one reason or any other reasons for queen replacement, including any other frequency of replacement.

#### 3.4.2. Replacement of Brood Comb

Of 108 respondents, 22 (20.4%) did not replace brood comb at all, and the same number replaced 31–50%, while replacement of 1–30% of comb was much more common (64 beekeepers; 59.3%), therefore a large majority replaced at least some brood comb per colony in 2017.

#### 3.4.3. Varroa Monitoring and Treatment, Resistance to Varroa, and Deformed Wing Virus

**Figure 5:**
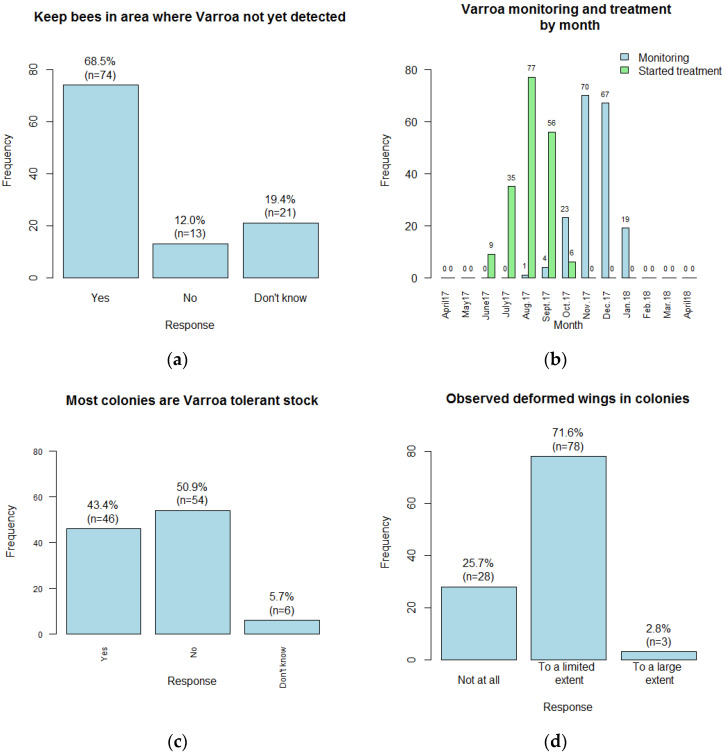
(**a**) Number of beekeepers stating whether they kept their bees in an area where Varroa had not yet been detected; (**b**) Number of beekeepers monitoring Varroa levels and starting Varroa control treatment each month; (**c**) Number of beekeepers stating that the majority of their colonies were Varroa-tolerant stock; (**d**) Numbers of beekeepers observing crippled or deformed wings in their colonies. Stated percentages may not add to 100% due to rounding error.

A high proportion, 68.5% (74 out of 108) of beekeepers, stated that their apiary was in an area where Varroa had still not been detected, while more beekeepers were unsure of the presence of Varroa than said “no” to this question (Figure 5a).

Monitoring of Varroa levels was done by 100 of 108 beekeepers (92.6%; Table 5). Although the percentages monitoring do differ according to whether or not Varroa was thought to have been detected in the area (*p* < 0.001), monitoring was done by almost all (73 of 74) of those saying that Varroa was not yet in their area as well as by most of the others. Beekeepers monitored most often in November or December, and reasonably often in October or January (Figure 5b).

Treating against Varroa was done by 95 of 108 (88.0%) beekeepers. Again, the proportions treating differed for different categories of response to whether or not Varroa was thought to have been detected in the area (*p* < 0.05), and again treatment was used by most beekeepers in each category, even by most (69 out of 74) of those thinking that Varroa was not present, which is surprising.

Beekeepers were asked whether they had Varroa-tolerant bees. Beekeepers whose bees appear to be resistant to Varroa may be less likely to treat against Varroa. Of 106, 46 (43.4%) claimed to have Varroa-resistant bees (Figure 5c), while more (54, 50.9%) stated that they did not, and 6 did not know. This is quite a high proportion claiming to have Varroa-resistant bees, yet 39 out of 46 (84.8%) of those thinking their bees were Varroa-resistant still treated against Varroa (Table 6), though not such a high proportion as for those who did not think this (*p* < 0.01).

While beekeepers were asked about a wide variety of possible Varroa control measures, the only Varroa control treatment carried out was use of tau-fluvalinate, as Apistan strips, most often beginning treatment in July, August or September (Figure 5b).

About one quarter of beekeepers claimed not to have observed deformed wings at all in their colonies, but the great majority had observed this to at least a limited extent (Figure 5d).

#### 3.4.4. Other Management Practices

**Figure 6:**
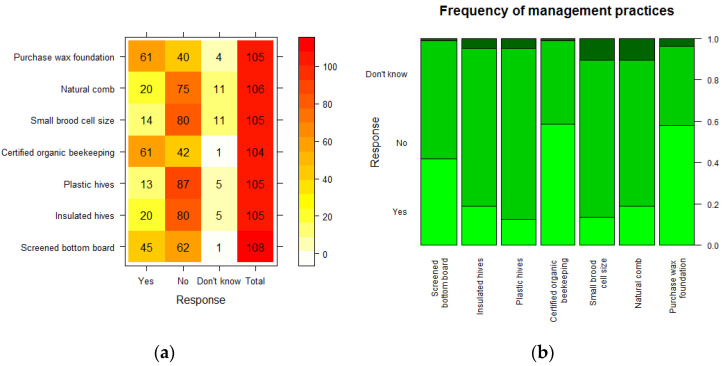
(**a**) Heat map of number of beekeepers using various management practices; higher numbers are indicated in red, lower numbers in yellow or white; (**b**) Relative proportions of “Yes”, “No” and “Don’t know” responses for each management variable.

Various other questions concerned topical issues currently addressed in the COLOSS questionnaire, to find out if any of these were relevant in the Saudi context. These questions were, for most of their beekeeping: whether or not the beekeeper used a screened bottom board (open mesh floor), insulated hives, or plastic hives (made of man-made materials), whether their beekeeping was certified organic, whether they used small brood comb size (5.1 mm cell size or smaller) or natural comb (without foundation), and whether they purchased wax-coated foundation. Figure 6 shows these results.

There were a higher number of “Don’t know” responses concerning use of natural comb and small brood cell size, and these questions may have needed more explanation. Only 14 of 105 respondents (13.3%) said they used small brood comb. The high proportion (75 of 106, 70.8%) not using natural comb is likely to reflect use of man-made hives rather than traditional beekeeping, while the high proportion (87 of 105, 82.9%) not using hives made of synthetic material is consistent with use of wooden hives or traditional hives. However, synthetic hives are more modern and 13 of 105 (12.4%) beekeepers did claim to use them. Insulated hives were mostly not used (80 of 105 (76.2%) did not use them compared to 20 of 105 (19.0%) who did), which is perhaps surprising in such a hot climate. For most of these questions in fact, “No” was the most common response, including for use of a screened bottom board, although these responses were more balanced (62 of 108 (57.4%) did not use these, as a Varroa control measure, while 45 of 108 did (41.7%)). There are arguments for and against these. A relatively high proportion purchased wax foundation (61 of 105; 58.1%), which is convenient and again consistent with use of man-made hives. A majority (61 of 104; 58.7%) said they practised certified organic beekeeping.

Finally, no respondent felt the need for additional training, and none reported having any concerns about their beekeeping other than issues already addressed in the survey.

## 4. Discussion

### 4.1. Apiary Environment and Migration

We obtained results concerning a wide range of aspects of the beekeeping of the participants in this beekeeper survey, including colony losses. A suitable environment as well as good management are important for colony survival. Most beekeepers kept their colonies in a shaded environment, as a means of protection against the extreme weather conditions, and over 90% migrated their colonies, most probably for cooler conditions and better forage availability [24].

While participants were asked about colony migration, they were not asked specifically whether this was for the purpose of pollination. For future work, collecting data on the participation of beekeepers in the pollination of agricultural crops would be worthwhile, as information about the intensity of agriculture in beekeeping areas could help to explain the loss of bee colonies. The latter would require detailed information on where a beekeeper’s colonies were located, and also on the location of where exactly colonies were moved to if they were migrated. Alternatively, questions could be included concerning the beekeeper’s own knowledge of crops in the vicinity of their apiaries. In addition to addressing the nature and diversity of agricultural crops, questions could be included on forage sources and availability.

### 4.2. Bee Race and Honey Yield

Concerning bee race, most beekeepers mainly kept local hybrid bees or the Yemeni honey bee. While the Yemeni bee is native to Saudi Arabia and thought to be better adapted to the local conditions than non-native bees [26,27,28,29,30,31], the Italian bee is considered as a better honey producer and it and the Carniolan bee *A.m. carnica* are often imported [24,32], so it is surprising to find only one beekeeper in this sample managing *A.m. ligustica* and none keeping *A.m. carnica.* The means to compare honey yields in this case was therefore limited; however, there was little difference in mean or median yields between local hybrid bee colonies and *A.m. jemenitica*. Honey yield per colony did greatly vary between beekeepers, from 0.839 kg to 7.937 kg, with a median of 1.379 kg and a mean of 1.604 kg. These averages are very low compared to values cited elsewhere in the literature [8,29,33,34].

Only 1 beekeeper indicated having more than one apiary, however, the number of colonies varied from 135 to 1700, with an average number of colonies exceeding 500 in all seasons. The very high numbers of colonies managed in a single apiary is likely to explain the low honey yield, although is not uncommon in apiaries of traditional hives [9]. A practical implication is that the larger-scale beekeepers should consider splitting their colonies between different smaller apiaries for better forage availability and higher production yield, where suitable alternative locations can be found and their management is feasible. Consideration of how to improve honey yield generally would be useful. Beekeepers should be supported in taking measures to increase yield by the beekeeping associations.

### 4.3. Levels of Colony Loss and Varroa Control

Over 80% of beekeepers reported colony losses, and in every season studied. As a country without an especially cold winter, colony losses in Saudi Arabia are relevant all year round. The overall colony loss rates were 11.43% (95% CI 10.21–12.77%) in summer 2017, 8.27% (95% CI 7.31–9.34%) in autumn 2017, 7.56% (95% CI 6.70–8.51%) in winter 2017/18 and 7.72% (95% CI 6.89–8.64%) and 6.59% (95% CI 5.82–7.44%) in spring 2017 and spring 2018, respectively. These are low levels of colony loss in all seasons compared to those in many countries. For example, the comparable national loss rates in the USA for summer 2017 and winter 2017/18 were 17.9% and 31.1% [2] and the winter 2017/18 loss rates for 36 countries in the COLOSS survey ranged from 2.0% to 32.8%, with a loss rate over all countries of 16.4% [19].

The loss rate is significantly higher in summer, a season when the weather conditions in Saudi Arabia are especially challenging due to the most extreme temperatures being experienced then, and the dry conditions limiting forage availability. Despite this, the beekeepers mostly attributed the losses to Varroa and disease, with weather much less often mentioned. This does seem inconsistent with the proportion of beekeepers believing Varroa was not present in their area, which was high, at nearly 70%, as Varroa is known to be present in the country [30,35]. Monitoring Varroa levels is advisable even if Varroa is not thought to be present, so that it does not take hold in a colony. In fact, 92.6% stated that they did monitor Varroa levels, including most of those not believing Varroa was in their area. Nearly 90% treated against Varroa, again including most of those not believing Varroa was in their area, which was unexpected. About 43% of beekeepers claimed to have Varroa-tolerant bees, yet about 85% of those still treated against Varroa. Colonies which show resistance to the Varroa mite need little or no treatment against Varroa, and such colonies which can co-exist with the Varroa mite have been identified in various countries [36]. There is strong current interest in the research community in breeding bees resistant to pests and disease [37], often native bees well-adapted to local environmental conditions including weather conditions and availability of forage. Equally, most of the beekeepers in this study were mainly keeping either local hybrid bees or the Yemeni bee, which are likely to be well-adapted to the environment and may also be tolerant of Varroa [38,39].

About three quarters of the respondents had observed deformed wings to at least a limited extent. This is consistent with the presence of the Varroa mite, as Deformed Wing Virus is associated with a high level of Varroa infestation of a colony [40], but is a surprising finding since the majority of the beekeepers claimed that Varroa had not yet been detected in their area. It also suggests that the presence of Varroa is having an impact on the colonies. It seems likely that Varroa is more widespread in the colonies in the studied regions than the beekeepers believed.

Beekeepers in many countries use a wide variety of Varroa control measures [41,42,43], and treatment strategy has been linked to colony loss [16,44] as well as the level of Varroa infestation [45]. However, in this study all beekeepers only used tau-fluvalinate as Apistan strips. In the absence of Varroa mite resistance to the synthetic pyrethroid tau-fluvalinate, this should be an effective treatment strategy [46,47,48,49,50] and Apistan strips are simple to use. In addition to possible chemical build-up in brood nest wax and pollen, and adverse effects on the bees [46,51,52,53], tau-fluvalinate has complex effects on the levels of Deformed Wing Virus, which can increase after the start of treatment [54], and a high percentage of beekeepers here did report signs of Deformed Wing Virus. However, beekeepers not monitoring for Varroa would be well advised to do so, in the event that treatment is needed.

Overall, there is a clear need for greater awareness of Varroa, a need to monitor for Varroa and infestation levels, and for advice on optimal treatment strategies in the conditions of Saudi Arabia.

### 4.4. Queen Replacement

Concerning other management practices, a higher percentage of young queens has been associated with better colony survival in Europe and elsewhere [16,20,21,55,56]. In the current study, 85% of beekeepers did replace queens but most often in the case of a lost queen rather than regular replacement. However, queen problems were rarely cited as a cause of colony loss. Investigating colony losses in relation to queen replacement and the reasons for doing this is an area for further work and would confirm whether regular replacement should be promoted.

### 4.5. Nature and Arrangement of the Hive

Regular replacement of a proportion of the brood comb may be recommended as a means of limiting build-up of pesticides or disease in the hive [57] and/or to avoid cells becoming smaller over time from an accumulation of contaminants, leading to less brood and smaller young bees [58]. Liu et al. [59] found brood comb renewal to be a significant factor for reducing the odds of colony loss. Nearly 80% of beekeepers in this study did replace comb to some extent.

Concerning use of screened bottom boards, about 42% of beekeepers used these in their colonies. Solid bottom boards provide more insulation and contain any hive debris in the hive, but require cleaning. On the other hand, screened or open mesh bottom boards allow ventilation and circulation of air, as well as allowing Varroa mites falling off bees to drop through and out of the hive, so are a means of Varroa control, but also allow access by other pests such as wax moths. In Saudi Arabia, ventilation in the hot months is likely to be of benefit to the bees. A follow-up question might ask about use of screened bottom boards in different seasons.

Insulated hives offer bees protection against the outside temperatures, which in Saudi Arabia are challenging for bees [60], but only 19% of respondents used these. However, a different protection strategy against the heat is to keep bees in a shaded environment and most beekeepers did do that. Hives made from synthetic materials are lighter and easier to handle than wooden hives, but expensive and difficult to clean. In developing countries where beekeepers may build their own hives using available materials, use of synthetic hives was not expected to be common, and in this sample of beekeepers only about 12% used them. In fact, this response, together with the high proportion not using natural comb (about 71%) and those who did purchase wax foundation (about 58%), all suggest a high degree of use of wooden hives. However, purchasing beeswax-coated comb foundation from commercial sources is a potential source of chemical pesticides from earlier treatments in the commercial beekeeping operations supplying the wax [61,62] and some beekeepers may prefer to avoid them.

While we asked about use of insulated hives and synthetic/plastic hives, as in the COLOSS survey, we did not ask specifically about use of traditional hives versus modern man-made box hives [9], and this would be a useful question to add in any future studies. Adgaba et al. [33] and Al-Ghamdi and Adgaba [29] cited about 62% and over 70% of beekeepers, respectively, as using traditional hives, so this may be changing. However, the study in [63] found lower levels of Varroa mites in traditional hives compared to frame hives.

Using small rather than conventional brood cell size in purchased foundation or allowing the bees to create natural comb should lead to raising smaller bees, owing to restricted conditions for bee development [64]. For various reasons, smaller cell size may limit reproduction of the Varroa mite [65,66], giving healthier bees, although not all studies have found this [63,67,68]. Use of small cell size comb and natural comb are popular in more natural and organic beekeeping. However, use of small cell comb was not common in this sample of beekeepers (only about 13% used this).

### 4.6. Organic Beekeeping

In response to the question of whether or not they practised certified organic beekeeping, nearly 59% of beekeepers answering this said that they did. However, this is inconsistent with most of the respondents using Apistan for Varroa control, a chemical treatment. Therefore, there is some doubt concerning the reliability of the responses to this question about organic beekeeping. It would be useful to investigate the nature of any claimed certification. Organic products usually attract a higher price than non-organic equivalents, which may be a reason for beekeepers to avoid use of non-organic chemicals in their hives and to become certified for organic beekeeping in order to sell honey labelled as organic. Concern for the environment may be another reason to motivate organic beekeeping in some places.

Approaches to Varroa control should be reviewed for those aspiring to produce organic honey, and the local beekeeper associations and national cooperative association could be instrumental in promoting and advising on different approaches. It may be the case that the question about organic beekeeping was not well understood, and this should be reviewed for future surveys; additional explanation could be provided.

### 4.7. Beekeeper Training

The participating beekeepers did not feel any particular need for training, which contrasts with the conclusion of Al-Ghamdi et al. [8] that there is a strong requirement for training programmes to overcome poor management practices. Our results reported above strongly suggest that training concerning Varroa management and honey production would be beneficial, in agreement with [8]. There would be challenges in providing such training, such as how to reach beekeepers in remote and inaccessible locations, and also the many beekeepers who may not have ready access to information provided online, as this is an obvious way to provide remote training. Our experience was that beekeepers are best contacted via phone and then visited personally, which is limiting in designing training programmes. The number of responses submitted in our online survey was small.

### 4.8. Nature of the Questions concerning Colony Losses

The questions asked on numbers of colonies and colony losses were deliberately kept simple for this first survey of colony losses. Determining numbers of colonies is difficult in conditions and seasons in which active management of colony numbers takes place, as in this country with very hot temperatures for a large part of the year. It is also difficult to state exact numbers of colonies and colonies lost when the number managed may be very large, and unless careful records are kept. There are also different definitions possible of what is meant by a lost colony, such as dead colonies, colonies with unresolvable queen problems, or colonies lost to natural disaster, as used in the COLOSS surveys, e.g., [21], or theft. In this survey, we did not distinguish between colonies lost due to different causes and left the decision of what was a lost colony to the beekeeper to decide when providing the answers. Therefore, while this approach gave a first impression of colony loss rates across the seasons, more detailed and more specific questions could provide more accurate and comparable information to loss rates from other countries. Further research would be worthwhile in a more in-depth and detailed study of colony losses.

### 4.9. Sample Representativeness and Sample Size

It is unclear whether the beekeepers in this study are in fact a wholly typical representative sample of all beekeepers in Saudi Arabia. The details of beekeepers to contact were provided by the heads of the local beekeeping associations, or their representative, and their reasons for selecting these particular beekeepers are unclear. For example, they may have recommended beekeepers they felt were more likely to take part, those known better to them, those more readily contacted, or larger-scale beekeepers. However, identifying the sample in this way did make this survey feasible to do in the presence of challenging survey conditions. In the absence of access to records of beekeepers, a random sample, for example, could not be selected. The region Rijal Almaa was under-represented in the survey relative to other regions (Table S1) but did have one of the smallest beekeeping associations.

The achieved sample size was modest, at 109 beekeepers, however, this was a first survey. The face-to-face methodology used in this case also limited the sample size. Future surveys should aim to increase the sample size to represent a larger percentage of the beekeepers in Saudi Arabia (Table S1). This survey also focused on the southwest of Saudi Arabia; while most beekeeping takes place in that part of the country, as noted above, it would be useful to involve beekeepers in other regions also, as their experience may be different. However, this would either greatly increase the time needed for a face-to-face survey or would require more survey personnel.

### 4.10. Beekeeper Characteristics and Operation Sizes Represented

All contacted beekeepers were male, and mostly well-educated. Female beekeepers in Saudi Arabia are rare [69,70], but might have different experience. We also did not ask about age of the beekeeper, but this could also help to assess how representative the sample was. However, beekeeping experience ranged from 2 years to 45 years, a wide range of levels of experience. The participants’ beekeeping operations are large, comprising 135 to 1700 colonies, and a majority described themselves as semi-professional or professional beekeepers. In many countries hobbyist beekeepers commonly keep only a few colonies [15,16,43], and managing over 50 colonies would be regarded as semi-commercial or commercial beekeeping. Therefore, it would also be worthwhile trying to establish how common smaller-scale beekeeping is in Saudi Arabia and, if it is common, whether the experience of those beekeepers may differ from what is reported here, in relation to colony loss rates but also concerning management practices. In several other surveys, the colony loss rates for smaller-scale beekeepers, keeping no more than 50 colonies, have been found to be significantly higher than for larger-scale operators, most probably owing to different management practices, as in the international COLOSS surveys [15,16,17,18,19,20,21], the pan-European studies in [71,72], Chinese surveys [6], and, for winter loss rates, the monitoring surveys of the Bee Informed Partnership in the USA, e.g., [2,73,74], whereas in the USA summer colony loss rates are lower for smaller-scale beekeeping. There may also be differing colony loss rates for different scales of beekeeping in Saudi Arabia, and this is a consideration for further research.

## 5. Conclusions

This study advances knowledge concerning apiculture in the Middle East by reporting results of the first purpose-designed survey of beekeepers in Saudi Arabia to examine management practices and levels of colony loss. A major aim was to examine differences in seasonal loss rates of colonies. The survey asked about five seasons: two spring seasons, one summer, one autumn and one winter, and found that the loss rate was significantly higher in summer than other seasons, at 11.4% of production colonies. However, the reported loss rates were generally low. To confirm these findings, it would be useful to repeat the survey to examine patterns of seasonal losses in other years. It would also be useful to be able to do a comparison with seasonal loss rates in other countries where beekeeping is practised all year round and seasonal and annual loss rates are important. The results reported here provide benchmarks for results from future surveys in this and other countries with a similar climate and environment.

Varroa and disease were the most commonly cited reasons for colony loss. While beekeepers did not feel the need for training, and most beekeepers were using Varroa treatment, the Saudi beekeeping associations, at both national and regional level, could usefully disseminate information to raise awareness of Varroa, encourage Varroa monitoring and evaluate approaches to Varroa treatment, helping to reduce losses and giving greater potential to exploit the organic honey market. Reported honey yields per colony varied much more between beekeepers than between bee races managed by beekeepers in the survey, yet almost all beekeepers stated honey production as a purpose of their beekeeping. Optimal hive management generally could result in higher honey yields for Saudi beekeepers and higher incomes for beekeepers. These in turn could help to attract new beekeepers and contribute to supplying the strong domestic market for Saudi honey. A priority for the local beekeeper associations and national cooperative association is therefore to find effective ways to promote and advise on good practice and actively support beekeepers.

In this article, we have reported extensive results from the conducted survey. Further work will relate colony loss levels to potential risk factors from the available information in the dataset. Follow-up surveys would also be worthwhile to confirm findings and allow more in-depth study of colony losses. Future surveys should aim to increase the sample size to represent a larger percentage of the beekeepers in Saudi Arabia and provide a more national rather than regional representation of the country, as beekeeping experience and practice may vary between locations where it is practised across this very large country.

## Figures and Tables

**Table 1:** Numbers of production colonies across the seasons, per beekeeper: Q1 is the lower quartile and Q3 is the upper quartile.

	Minimum	Q1	Median	Mean	Q3	Maximum
Spring 2017	155	360.0	540.0	575.5	710.0	1600
Summer 2017	150	350.0	500.0	570.0	750.0	1500
Autumn 2017	149	350.0	500.0	559.0	700.0	1300
Winter 2017/18	140	380.0	500.0	556.9	700.0	1300
Spring 2018	135	400.0	540.0	579.4	720.0	1700

**Table 2:** Apiary environment and conditions.

Environment	Frequency	%	Conditions	Frequency	%
Forest/woodland	73	67.6%	Sunny	38	35.2%
Cultivated field	34	31.5%	Shaded	69	63.9%
Orchard	1	0.9%	Sheltered/closed place	1	0.9%
Total	108	100%	Total	108	100%

**Table 3:** Honey production in kg per beekeeper, using available data.

	Minimum	Q1	Median	Mean	Q3	Maximum	No. of Beekeepers
Overall total	200.0	575.0	800.0	889.4	950.0	5000.0	108
Overall, per colony	0.839	1.231	1.379	1.604	1.618	7.937	108
Local hybrids, per colony	0.909	1.247	1.404	1.604	1.603	7.937	78
*A.m. jemenitica*, per colony	0.839	1.222	1.340	1.619	1.923	3.906	28
*A.m. ligustica*, per colony	1.572	1.572	1.572	1.572	1.572	1.572	1

**Table 4:** Summary of numbers of colonies lost per beekeeper and loss rates with 95% confidence intervals (CIs), by season; Q1 and Q3 are lower and upper quartiles.

Season	Min.	Q1	Median	Mean	Q3	Max.	Total No. of Colonies	Total No. of Colonies Lost	% Colonies Lost (95% CI)
Spring 2017	0	20.0	40.0	44.4	70.0	150	62,725	4840	7.72 (6.89, 8.64)
Summer 2017	0	40.0	60.0	65.1	95.0	300	62,125	7100	11.43 (10.21, 12.77)
Autumn 2017	0	20.0	40.0	46.2	65.0	300	60,929	5037	8.27 (7.31, 9.34)
Winter 2017/18 *	0	20.0	40.0	42.1	65.0	200	60,150	4545	7.56 (6.70, 8.51)
Spring 2018	0	15.0	35.0	38.2	55.0	140	63,155	4160	6.59 (5.82, 7.44)

* Excluding one beekeeper with missing information for number of colonies.

**Table 5:** Monitoring and treatment of Varroa, against Varroa not yet detected in the area in which bees are kept.

		Monitor Levels of Varroa					Treat against Varroa				
**Varroa** **not detected in** **area**	**Response**	**Yes**	**No**	**Don’t Know**	**Total**	**%**	**Yes**	**No**	**Don’t Know**	**Total**	**%**
	Yes	73	1	0	74	68.5	69	5	0	74	68.5
	No	11	2	0	13	12.0	11	2	0	13	12.0
	Don’t know	16	1	4	21	19.4	15	5	1	21	19.4
	Total	100	4	4	108		95	12	1	108	
	%	92.6	3.7	3.7			88.0	11.1	0.9		

**Table 6:** Frequency of treatment of Varroa, against whether colonies are considered as Varroa-tolerant stock.

		Treat against Varroa				
**Varroa** **tolerant stock**	**Response**	**Yes**	**No**	**Don’t Know**	**Total**	**%**
	Yes	39	7	0	46	43.8
	No	50	3	0	53	50.5
	Don’t know	3	2	1	6	5.7
	Total	92	12	1	105	
	%	87.6	11.4	1.0		

## Data Availability

Not applicable.

## Supplementary Materials

The following supporting information can be downloaded at: https://www.mdpi.com/article/10.3390/insects14060513/s1, Questionnaire S1: Purpose-designed survey questionnaire used for data collection, Table S1: Number of beekeepers visited in each region and response rates.
