# Supplementary material for: Seasonal Colony Loss Rates and Honey Bee Management in the Kingdom of Saudi Arabia: Results of a Survey of Beekeepers
Source: Insects. 2023 Jun 1;14(6):513. doi: 10.3390/insects14060513 (PMC10298960; doi:10.3390/insects14060513)
Supplement: Supplementary file 1 [file insects-14-00513-s001.zip › insects-2360489-supplementary.pdf]

insects

MDPI

Supplementary Materials

Questionnaire S1. Purpose-designed survey questionnaire used for data collection.

Saudi Honey bee loss and survival survey 2017/2018

The notes E① etc. provide some essential information about the questions below.

About you as a beekeeper

E① Information about your name and address and personal details will be removed from the file used for processing the data and kept confidential.

First name ______________________
Last name ______________________
Address ______________________
City ______________________
Postal Code ______________________
Email ______________________
Phone number ______________________

1) What is your level of education? O primary school O middle school O secondary school O university O self-taught

2) What is your profession? ……………………………………………………………………

3) What kind of beekeeper are you?

O A hobby beekeeper
O A semi-professional beekeeper whose beekeeping provides a substantial part of their income
O A professional whose beekeeping is their main income?

4) How many years have you been keeping bees? (You may state part of a year, e.g. 0.5 for half a year).

5) What influenced you to become a beekeeper? ……………………………………………………………

6) Do you keep bees mainly to:
O produce honey O breed and sell bees (queens or nucleus colonies)
O both the above O Other (please specify) ………………………………………………

About your apiary

7) To describe the location of your main apiary or operation, please state

a) the name of a city/town/village near to your apiary? ____________

b) and the postal code of the apiary (or a postal code nearby ____________

8) What is the name of the region where you keep your bees? ____________

9) How many apiaries do you have? [ ]

10) If you have more than one apiary, are all your apiaries within a distance of about 15 km of each other? If you have only one apiary, please answer yes.

O Yes O No O Don't know

11) What is the environment of your main apiary like?

O Forest/woodland O Cultivated field O Orchard O Garden/yard

O Other (please specify)

………………………………………………………………………………

12) Is the location of your main apiary:

O Sunny? O Shaded? O Sheltered/closed place?

O Other? (please specify)

…………………………………………………………………………….....

About your colonies and colony losses

E② In this questionnaire we try to gather information about production colonies. *Please consider colonies which are queenright and strong enough to provide a honey harvest as production colonies.*

13) How many production colonies did you have in total during

Spring 2017: .... Summer 2017: .... Autumn 2017: ....

Winter 2017–2018: .... Spring 2018: ....

We are considering Spring as March to April, Summer as May to August, Autumn as September to October, and Winter as November to February.

E③ Please consider a colony as lost if it is dead (or reduced to a few hundred bees) or alive but with queen problems, like drone laying queens or no queen at all, which you couldn't solve.

14) Have you observed any losses of production colonies during the period from 2017 to 2018?

O Yes O No

14a) How many production colonies did you lose in total during

Spring 2017: .... Summer 2017: .... Autumn 2017: ....

Winter 2017–2018: .... Spring 2018: ....

We are considering Spring as March to April, Summer as May to August, Autumn as September to October, and Winter as November to February.

14b) If you lost colonies, what do you think are the main cause(s) of your colony losses from 2017 to 2018? You may specify more than one cause.

O Varroa? O Disease? O Queen problems? O Starvation?
O Extreme weather? O Other? (please specify) ....

About bee management and other matters

15) Did you migrate any of your colonies at least once for honey production or pollination or other reasons in 2017? O Yes O No

15a) If you answered Yes to 15, when did you move your bees and where did you move them?

....
....

16) What was your total honey production in kg over the past year? [ ]

17) Approximately what proportion of brood combs did you replace with comb foundation per colony in 2017?
O 0% O 1–30% O 31–50% O more than 50%

18) Do you keep your bees in an area where Varroa has still not been detected?
O Yes O No O Don't know

19) Have you monitored your colonies for Varroa during the period April 2017-April 2018?
O Yes O No O Don't know / not applicable

20) Have you treated your colonies against Varroa during the period April 2017 - April 2018?
O Yes O No O Don't know / not applicable

21) Could you please indicate the months when you monitored your production colonies for Varroa AND also indicate when you STARTED a Varroa treatment or management plan during the period April 2017 - April 2018? **Method / Product:**	Month in which each measure started: 2017 April	May	June	July	August	September.	October	November	December	2018 January	February	March	April
Monitoring of Varroa infestation level (e.g. counting mite fall)													
Drone brood removal													

Hyperthermia (heat treatment of brood/bees)													
Other biotechnical method (as e.g. trapping comb, complete brood removal, queen confinement)													
Formic acid - short term													
Formic acid - long term (e.g. MAQS)													
Lactic acid													
Oxalic acid - trickling													
Oxalic acid - sublimation (evaporation)													
Hiveclean/Bienenwohl/Beevital													
Thymol (e.g. Apiguard, ApilifeVar)													
Tau-fluvalinate (e.g. Apistan)													
Flumethrin (e.g. Bayvarol)													
Amitraz (in strips, e.g. Apivar, Apitraz)													
Amitraz (fumigation/aerosol)													
Coumaphos (e.g. Perizin)													
Coumaphos (in strips, e.g. Checkmite+)													
Another chemical product													
Another method													

22) Have you noticed bees with crippled/deformed wings in your colonies? (These are signs of the presence of Deformed Wing Virus, which is spread by Varroa mites).

O Not at all O to a limited extent O to a large extent O Don't know

23) Which of the following measures apply for the majority of your beekeeping:

a) Screened bottom board O Yes O No O Don't know
b) Insulated hives O Yes O No O Don't know
c) Plastic hives O Yes O No O Don't know
d) Certified organic beekeeping O Yes O No O Don't know
e) Varroa tolerant stock O Yes O No O Don't know
f) Small brood cell size (5.1 mm or less) O Yes O No O Don't know
g) Natural comb (without foundation) O Yes O No O Don't know
h) Purchase wax from outside own operation O Yes O No O Don't know

24) Are there pests which are a threat to the honey bees in your apiary/apiaries?

O Yes O No O Don't know

24a) If you answered Yes to 24, what are these pests?..

....

....

25) Do you protect your colonies from the weather? O Yes O No

25a) If you answered yes to 25, which methods do you use?

....

……………………………………………………………………………………………

……………………………………………………………………………………………

26) What strain/race of bees do you mostly keep in your apiary/apiaries?

O Local hybrid of no specific race O The Yemeni honey bee

O The Carniolan honey bee O The Italian honey bee

O Other race (please specify which one)

....

27) Do you replace queens? O Yes O No

27a) If your answer to 27 was yes, in which case do you replace queens?

O Only in case of loss of queen

O Replaced yearly

O In case of poor egg laying by the queen

O Other (please specify).

....

28) Do you feel that you have any needs for training or support in your beekeeping?

28a) If you answered yes to 28, what are these training needs? ....

29) Are there are any other concerns that you have in your beekeeping which have not been asked about above? If yes, please say what these are:

....

....

Thank you very much for your time in answering these questions. We hope that they will give a useful picture of beekeeping in Saudi Arabia and in particular that by studying colony losses, some possible risk factors for colony loss may be identified and followed up.

Table S1. Number of beekeepers visited in each region and response rates.

**Region visited**	**Dates of visits**	**No. of members**	**No. of contacts (% of members)**	**No. visited (% of members)**	**% of contacts visited**	**Total no. of respondents (visits and online)**	**% of beekeepers in survey**
Makkah	10/05/2018 to 20/05/2018	88	20 (22.7%)	13 (14.8%)	65.0%	15	13.8%
Taif	21/05/2018 to 03/06/2018	56	22 (39.3%)	16 (28.6%)	72.7%	17	15.6%
AlBaha	04/06/2018 to 17/06/2018	115	20 (17.4%)	15 (13.0%)	75.0%	21	19.3%
Abha	18/06/2018 to 01/07/2018	47	21 (44.7%)	17 (36.2%)	80.9%	19	17.4%
Rijal Almaa	02/07/2018 to 15/07/2018	32	15 (46.9%)	7 (21.9%)	46.6%	7	6.4%
Jazan	16/07/2018 to 29/07/2018	60	19 (31.7%)	14 (23.3%)	73.6%	14	12.8%
Najran	30/07/2018 to 12/08/2018	29	24 (82.8%)	16 (55.2%)	66.6%	16	14.7%
Total	-	427	141 (33.0%)	98 (23.0%)	69.5%	109	100.0%
